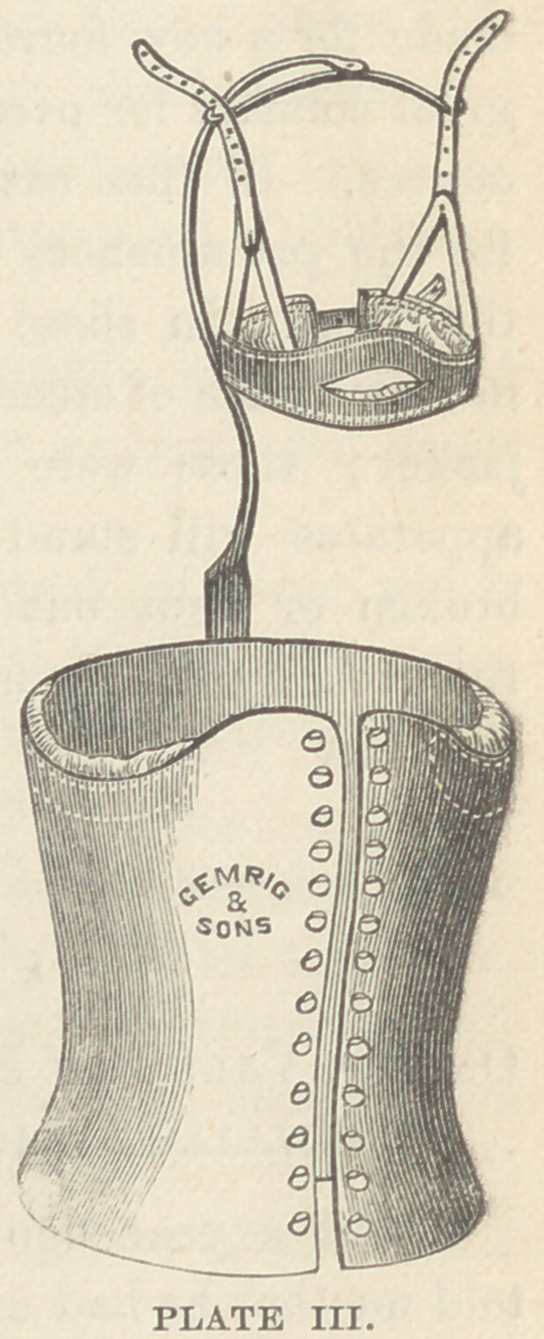# On the Mechanical Treatment of Pott’s Disease

**Published:** 1880-03

**Authors:** Geo. F. Souwers

**Affiliations:** Philadelphia


### ARTICLE IV.

### ON THE MECHANICAL TREATMENT OF POTTS' DISEASE. A New Apparatus by Mr. Gemrig, of Philadelphia. By Dr. GEO. F. SOUWERS, Philadelphia.

There is, perhaps, in the wide domain of surgery no disease the treatment of which has of late years attracted so much attention as caries of the spinal vertebræ, or, to employ the name commonly given the trouble, Potts' disease of the spine. Blisters, cauteries and various bandages and devices have rapidly followed each other; each has had its day, served its purpose, and been relegated to a greater or less degree of oblivion. It may not be uninteresting to the general practitioner, and to those not in the neighborhood of the large cities and hospitals, to consider some of the different forms of apparatus, etc., that have been and are still used.

In the early stage of the disease, before deformity has become particularly noticeable, the best form of apparatus, probably, is the bed. The patient should be placed in the recumbent position on a strong hair mattress, from which a U-shaped piece has been removed, for the easier introduction of a bed-pan; this position should be maintained until either the patient is fairly recovered, or until it becomes evident that more decided measures are necessary for his relief. As to the employment of cauteries, etc., in this stage we shall say nothing, this paper having only to do with the purely mechanical treatment. Probably the simplest and cheapest form of apparatus—when the stage for mechanical interference arrives—is the plaster jacket, so much employed by Dr. Sayre, of New York; it presents the advantages of support to the yielding column and readiness of application. The method of making and applying this jacket is as follows: Into the meshes of coarse crinoline or Swiss muslin, dry plaster of Paris is rubbed, the crinoline being in strips about two or three inches in width; this is made into an ordinary roller bandage, which should be put away in an air tight box till needed.

When it is desired to use the bandage it is dipped *end* first into cold water and applied as follows: A thin close fitting gauze sleeveless shirt, having loops to tie over the shoulders, is placed on the patient, the bandage is then carried round the body, each lap covering one half the previous turn; over the stomach a pad is placed, which can be drawn out before the plaster has thoroughly set; over the laps of the bandage wet plaster is rubbed, and the whole is then allowed to dry.

The patient while being bandaged should be suspended, either by bands passing under the axilla, or by a halter catching the chin and occiput. A very convenient apparatus for this purpose is here figured (plate I); or a hook having been driven into the ceiling answers the same purpose. In an institution with which I am connected, a beam of wood is placed between the ends of two book-cases, which occupy a corner, and from the beam an ordinary block and tackle is suspended. For the purpose of securing greater supporting power, narrow strips of tin are placed around the body at intervals of two or three inches, after two or three layers of the bandage have been applied. The chief objection to the plaster bandage is its liability to crack, and in places to chip off; this should of course be watched and remedied as soon as it happens. Further, ablution is not as readily performed as is desirable, and this of course is objectionable; though taking the bandage as a whole, its good far outweighs its evil features. Without any discomfort it may be worn for months, if so desired.

PLATE I.

Another form of this bandage is the silicate dressing, the principles of which are the same, to wit, a strong, supporting, unyielding box, which shall act as it were as an external spinal column. Stiff binder's board, wet and moulded to fit the body, or strong felting, will also answer a good purpose.

In those cases where there is but a slight tendency to spinal curvature, a jacket such as is here shown (plate II) answers a

PLATE II.

good purpose. The lower or abdominal portion of the apparatus being elastic, whilst the thoracic portion laces up. The weight of the head and shoulders is transferred by two crutch heads, which fit into the axilla through a steel straight spring to the hips. In cases of lateral curvature, a somewhat similar apparatus, to which a pad has been added, as figured below (plate III), may be employed. As a support in posterior curvature, the apparatus here depicted has some advantages. A pelvic band supports two lateral crutches, which reach up to the axilla. Posteriorly, an upright fitted with an axillary strap extends up on either side of the spinal column.

There are, of course, many other and varied forms of apparatus, but it is a question whether the instrument lately contrived by Mr. Gemrig, of Philadelphia, and described below, does not present greater advantages than any now in use. This apparatus, which has been very successfully employed in the Pennsylvania Hospital, in Philadelphia, and the manner of making it, may be described as follows: An accurate mould of the trunk is taken by means of the plaster jacket, the patient of course being suspended; before becoming entirely set the jacket is cut open in front, in the middle line. Into the mould thus obtained a quantity of mixed plaster is poured, and this on hardening presents a perfect cast of the suspended body. A

PLATE III.

piece of thick, half tanned leather, thoroughly saturated, is now applied to the cast thus obtained, and having been accurately molded into position, is allowed to dry. Upon examination after drying, the cast is found to be so accurate that even the intercostal and intermuscular spaces are clearly shown in the leather. The dried jacket is now lined with fine chamois skin, cushioned at the axillary edges, and fenestræ cut in it, thus providing for ventilation, which the plaster jacket, with all its advantages, does not possess. Eyelets and lacing cords are now fitted in front, and the apparatus is complete. If desirable, the apparatus may be removed at night, the mattress then acting as the spinal support. To this apparatus Dr. Sayre's "jury-mast" may be added; this supports the head in an erect position, and the spine is relieved of superincumbent weight by a rod reaching above the back of the head, and from which supporting bands extend beneath both occiput and lower jaw.

This jacket is not expensive, and when the plaster jacket has been applied, a leather jacket can readily be fashioned from it. Its weight is almost nil comparatively. I have before me a jacket made for a boy fourteen years of age, and worn by him with great comfort for over six weeks, the weight of which is eighteen ounces. In this case fenestræ were cut in the jacket, to allow for the prominences of the anterior superior spinous processes of the ilium. In those cases in which extra support is required, narrow bands of steel may be placed on the back and sides of the jacket; these were used in the case above mentioned. The apparatus will stand a large amount of ill-usage and not be broken or bent out of position. The jacket can readily be removed by simply unlacing the front, thus providing for ablution etc.

## Figures and Tables

**PLATE I. f1:**
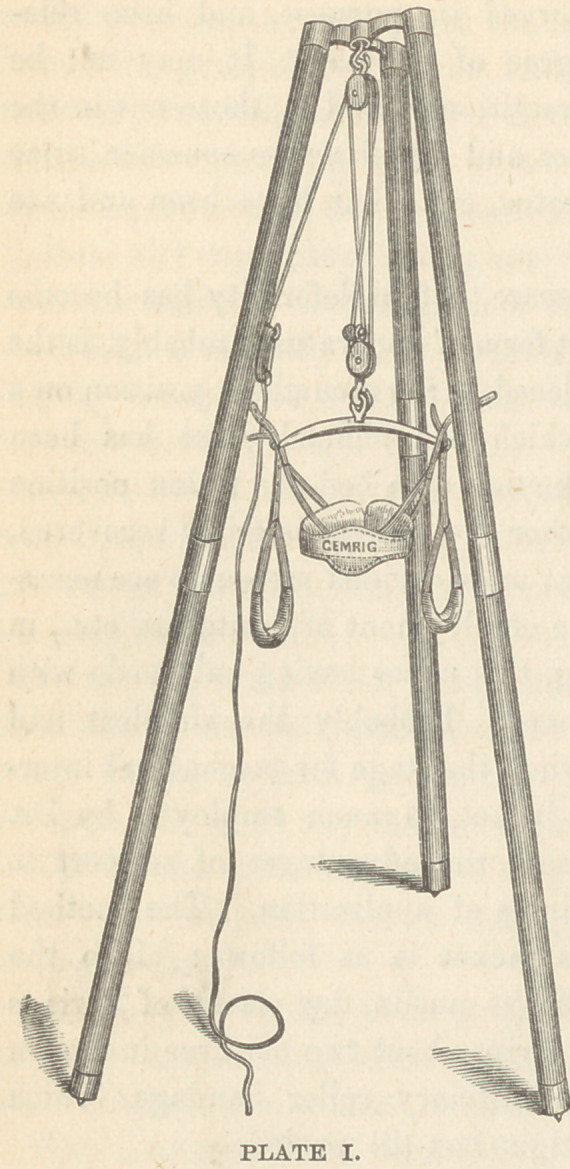


**PLATE II. f2:**
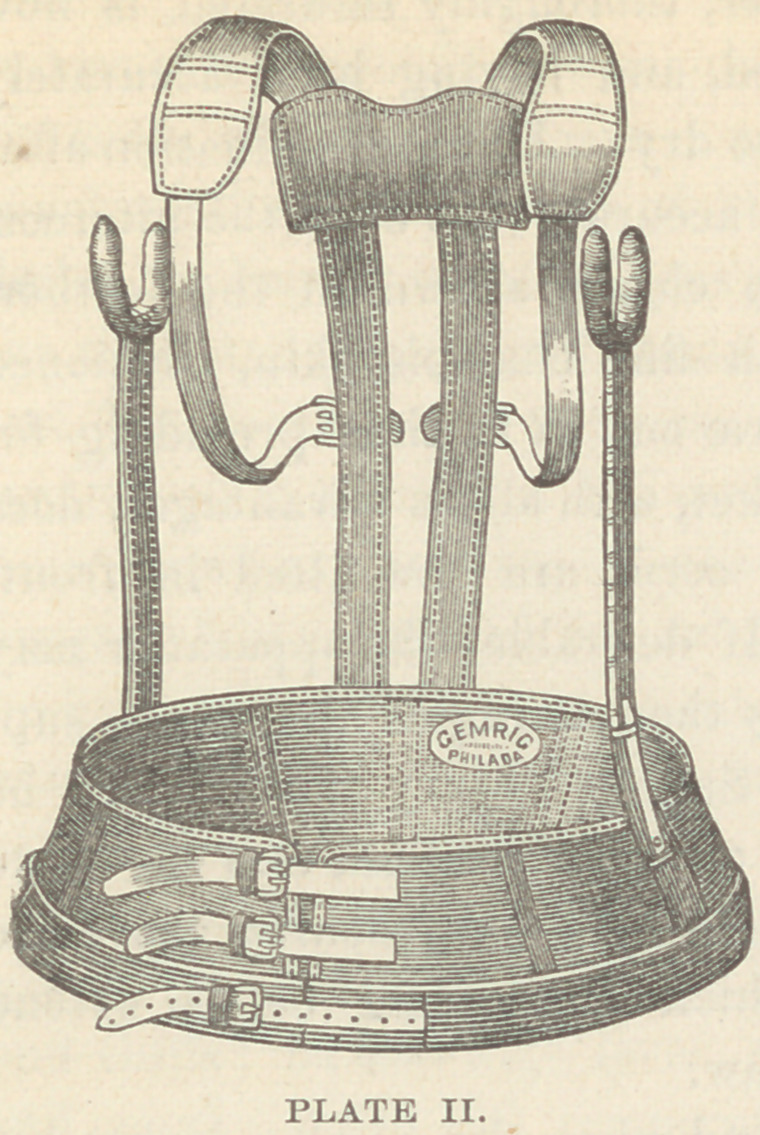


**PLATE III. f3:**